# Six out of ten patients with sinus tarsi syndrome returned to pre-injury type of sport after subtalar arthroscopy

**DOI:** 10.1007/s00167-020-06385-8

**Published:** 2020-12-19

**Authors:** Kenny Lauf, Jari Dahmen, J. Nienke Altink, Sjoerd A. S. Stufkens, Gino M. M. J. Kerkhoffs

**Affiliations:** 1 Department of Orthopedic Surgery, Amsterdam Movement Sciences, Academic Medical Center, Amsterdam UMC, Location AMC, University of Amsterdam, Meibergdreef 9, 1105 AZ Amsterdam, The Netherlands; 2 Academic Center for Evidence Based Sports Medicine (ACES), Amsterdam UMC, Amsterdam, The Netherlands; 3 Amsterdam Collaboration for Health and Safety in Sports (ACHSS), International Olympic Committee (IOC) Research Center, Amsterdam UMC, Amsterdam, The Netherlands

**Keywords:** Sinus tarsi syndrome, Arthroscopic treatment, Ankle, Subtalar arthroscopy, Return to sport

## Abstract

**Purpose:**

The purpose of this study was to determine multiple return to sport rates, long-term clinical outcomes and safety for subtalar arthroscopy for sinus tarsi syndrome.

**Methods:**

Subtalar arthroscopies performed for sinus tarsi syndrome between 2013 and 2018 were analyzed. Twenty-two patients were assessed (median age: 28 (IQR 20–40), median follow-up 60 months (IQR 42–76). All patients were active in sports prior to the injury. The primary outcome was the return to pre-injury type of sport rate. Secondary outcomes were time and rate of return to any type of sports, return to performance and to improved performance. Clinical outcomes consisted of Numerous Rating Scale of pain, Foot and Ankle Outcome Score, 36-item Short Form Survey and complications and re-operations.

**Results:**

Fifty-five percent of the patients returned to their preoperative type of sport at a median time of 23 weeks post-operatively (IQR 9.0–49), 95% of the patients returned to any type and level sport at a median time of 12 weeks post-operatively (IQR 4.0–39), 18% returned to their preoperative performance level at a median time of 25 weeks post-operatively (IQR 8.0–46) and 5% returned to improved performance postoperatively at 28 weeks postoperatively (one patient). Median NRS in rest was 1.0 (IQR 0.0–4.0), 2.0 during walking (IQR 0.0–5.3) during walking, 3.0 during running (IQR 1.0–8.0) and 2.0 during stair-climbing (IQR 0.0–4.5). The summarized FAOS score was 62 (IQR 50–90). The median SF-36 PCSS and the MCSS were 46 (IQR 41–54) and 55 (IQR 49–58), respectively. No complications and one re-do subtalar arthroscopy were reported.

**Conclusion:**

Six out of ten patients with sinus tarsi syndrome returned to their pre-injury type of sport after being treated with a subtalar arthroscopy. Subtalar arthroscopy yields effective outcomes at long-term follow-up concerning patient-reported outcome measures in athletic population, with favorable return to sport level, return to sport time, clinical outcomes and safety outcome measures.

**Level of evidence:**

IV.

## Introduction

The sinus tarsi is the space between the talus and calcaneus on the lateral side of the foot. Relevant ligamentous structures in the sinus tarsi are the cervical ligament (CL), the interosseous talocalcaneal ligament (ITCL) and the inferior extensor retinaculum (IER). In case of a (partial) rupture of the ITCL, hindfoot instability could be experienced. This hindfoot instability in addition to experiencing pain on the lateral side of the ankle is the most common combination of complaints in people diagnosed with sinus tarsi syndrome (STS) [3, 11–13, 15, 22]. Other factors, such as impingement, an inflammatory condition, a loose body, a (partial) tear of the CL and a deformity of the foot, are described in literature as cause of STS [11, 13].

When patients are diagnosed with sinus tarsi syndrome, a number of treatment options are available. Local injections with anesthetics or corticosteroids, physiotherapy with appropriate proprioception training or an immobilization period can be part of the conservative treatment algorithm [4, 9, 22]. In up to 60% of patients conservative treatment is clinically effective [22]. However, when complaints are persistent over time, an operative intervention—being an open or arthroscopic procedure—may be considered [15].

Concerning the evidence on the efficacy of operative interventions for STS, it is clear that a low number studies with limited number of included patients and low methodological quality have been published [12, 17, 18]. Oloff et al. [18] reviewed 29 cases of patients treated with subtalar arthroscopies as diagnostic or therapeutic treatment for sinus tarsi syndrome. Lee et al. [12] published a study of 33 patients regarding arthroscopic findings and clinical outcomes. A similar study with 8 patients has been performed by Mansur et al. [17]. No long-term follow-up study has been performed up to date for the case of subtalar arthroscopies for sinus tarsi syndrome. Moreover, sports outcomes with specific included levels of return to sport, return to play and return to performance are absent in the literature on STS treatment [2]. Consequently, there is an inadequate amount of evidence on the efficacy of a subtalar arthroscopy procedure for STS with specific sports and clinical outcomes at long-term follow-up.

Hence, it is the purpose of the present study to assess the long-term sports and clinical outcomes of subtalar arthroscopy as treatment option for sinus tarsi syndrome. It was hypothesized that this specific treatment option would provide satisfying sports and clinical outcomes at a long-term follow-up. With the first available sports outcomes in literature, the present study has the potential to set a reference frame for athletes regarding the long-term effect of subtalar arthroscopies as treatment option for sinus tarsi syndrome. This reference frame is important for (elite) athletes when the treating clinical team is considering different treatment options that are available.

## Materials and methods

**Fig. 1:**
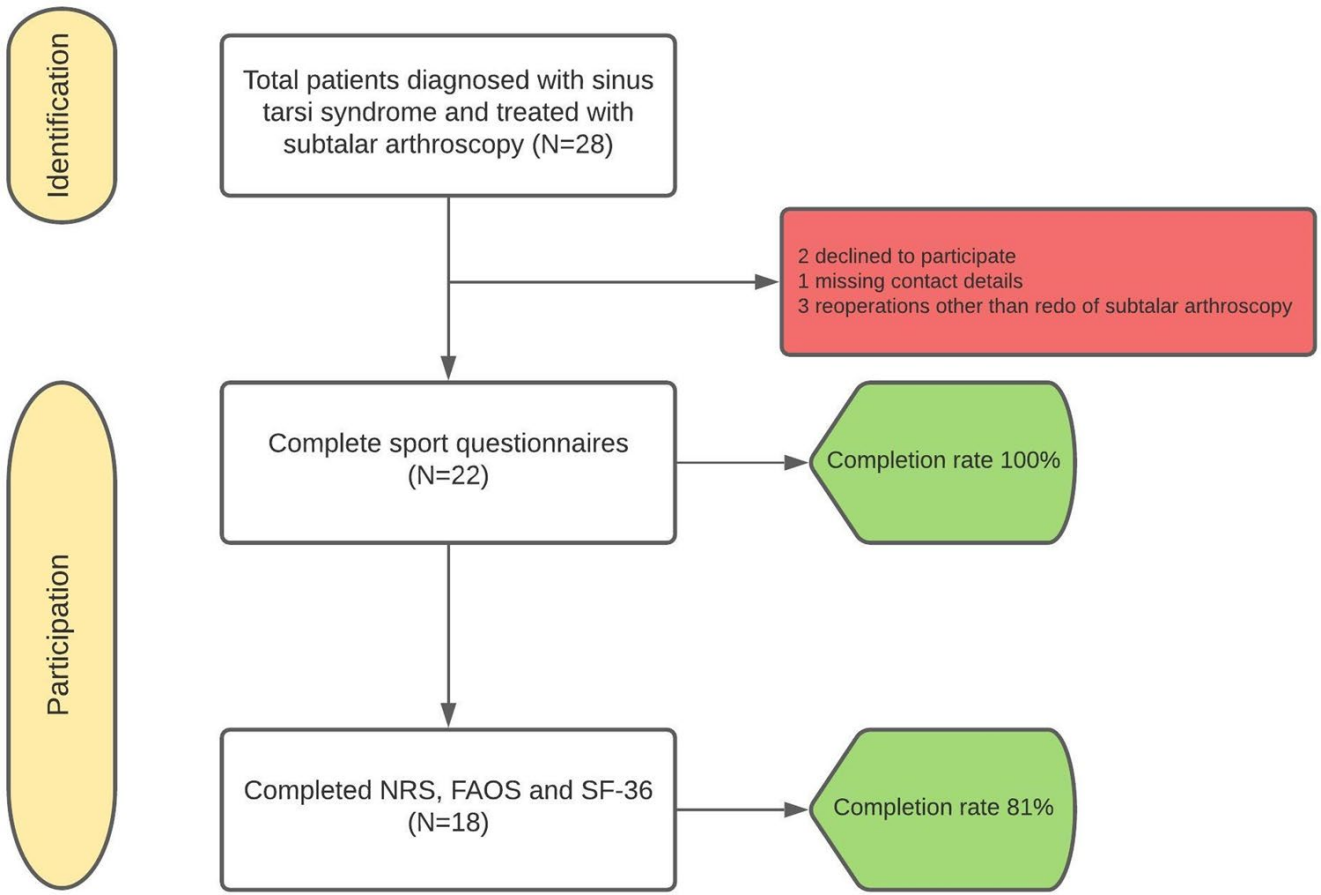
Flowchart of participation

The study was approved by the local Medical Ethics Committee of the Amsterdam University Medical Centre with reference number MEC 08/326 and was performed in accordance with the principles of the Declaration of Helsinki and the medical Research Involving Human Subjects Act (WMO). All patients treated between 2013 and 2018 for sinus tarsi syndrome with a subtalar arthroscopy by two experienced fellowship-trained foot and ankle orthopedic surgeons (GK & SS) of a large academic tertiary referral center were included. Sinus tarsi syndrome was defined as palpable pain over the sinus tarsi with or without concomitant subjective symptoms of ‘giving way’. A preoperative Magnetic Resonance Imaging (MRI) scan was performed to determine the causing substrate for complaints as well as the location of the affected tissue(s). Indications for surgery in patients with sinus tarsi syndrome were the combination of a failed conservative period of at least 1 year and the presence of one or more loose bodies, impingement of soft tissue or osseous structures, ganglions and synovitis in the sinus tarsi. Exclusion criteria are posted in Table 1. A total of 28 patients who underwent subtalar arthroscopy for sinus tarsi syndrome between January 2013 and December 2018 were identified and contacted (Fig. 1). A total of 22 patients were benevolent for participation in this study. There were nine males and thirteen females. The median age at surgery was 28 years (IQR 20–40 years) and the mean follow-up time was 60 months (IQR 42–76 months).

**Table 1:** Exclusion criteria

Exclusion criteria
Follow-up < 1 year
Conservative treatment for sinus tarsi syndrome
Re-operations other than re-do subtalar arthroscopy after index subtalar arthroscopy
Patients with symptomatic chronic lateral ankle instability requiring surgery

### Operative technique

**Fig. 2:**
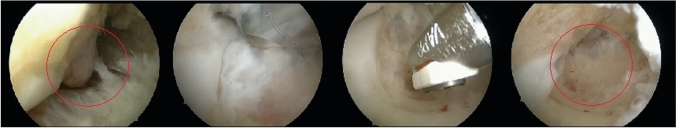
Perioperative arthroscopic images. Arthroscopic images during removal of soft-tissue impingement in the sinus tarsi. From left to right the: the impinging soft tissue (marked), the use of the shaver, the use of the VAPR and the sinus tarsi after removal of the impinging soft tissue (marked)

All procedures were carried out under spinal or general anesthesia and in supine position. A small cushion was placed on the ipsilateral side and a tourniquet was used on the ipsilateral thigh. The sinus tarsi was palpated after which the first arthroscopic portal, the anterior portal, was introduced 1 cm inferior and 1–2 cm anterior to the lateral malleolus—the posterior portal was placed under direct arthroscopic visualization of the anterior portal 1 cm inferior of the distal tip of the lateral malleolus. These two portals were used in an alternating fashion for visualization and for instrumentation use. The subtalar joint was inspected for intra-articular pathologies: the calcaneofibular ligament, interosseous ligament, cervical ligament, cartilage of the subtalar joint and other anatomical structures were examined. When necessary, the shaver and/or the VAPR (Radiofrequency) was used for soft-tissue impinging synovectomies, fibrous soft-tissue ablation, and joint debridement [5]. In case of presence of a ganglion, a ganglionectomy was performed [16]. Ganglion cysts are synovial cysts filled with gelatinous mucoid material. Although the exact etiology of the development of ganglion cysts is unknown, they are believed to arise from repetitive microtrauma resulting in mucinous degeneration of connective tissue [7]. A grasper was used for loose body removals. When a non-impinging joint condition was reached without the presence of synovitis nor ganglions, both the articular cartilage of subtalar joint as well as subtalar motion were examined under direct arthroscopic visualization. After closure of soft-tissue layers and skin, a pressure bandage was applied when the procedure was finished. In Fig. 2, the arthroscopic procedure of soft-tissue impingement removal is presented.

### Postoperative planning

The compression dressing was advised to be worn for 2 days postoperatively. Full weight-bearing was allowed directly postoperatively. However, in case of discomfort and too much pain, the patient was allowed to use crutches for the first few days. Two weeks postoperatively, the sutures were removed in the outpatient clinic and the patient was instructed to initiate active rehabilitation of passive as well as active range of motion—with focus on subtalar joint motion and ankle motion—and strength exercises. At 6 weeks postoperatively, a clinical evaluation was performed to assess the clinical efficacy of the surgery and to check assess whether a gradual protocol-based build-up towards returning to activity, sport and performance was permitted under supervision of an experienced physiotherapist.

### Study procedure and data collection

All eligible patients were extracted from our electronic patient file system and all patients were contacted and invited to participate in the study. After patients’ approval, sports outcomes were retrieved through a phone call and patient-reported outcome measures (PROMs) reflecting the clinical outcomes were obtained through an electronic questionnaire (Castor EDC). When no response was recorded, two reminder emails and phone calls were executed. Re-operation, complication and demographic data were collected from the electronic patient system.

### Outcome assessment

The primary outcome was defined as the ‘return to pre-injury type of sport’ rate of the total of the study population. All outcomes retrieved from the patient population are summarized in Table 2.

**Table 2:** Overview of the primary and secondary outcomes

Primary outcome
Return to pre-injury type of sport rate
Secondary outcomes
Demographic factors
Sports outcomes Level of sport Return to any level and any type of sport rate—excluding the rehabilitation process Return to performance rate—defined as pre-injury level and type of sport Return to improved performance rate—improvement of level of sport in comparison to pre-injury level Return to sport time at above-mentioned levels (number of weeks postoperatively) Clinical outcomes PROM: Numeric rating scale of pain in rest, during walking, during running and stairclimbing PROM: Foot and Ankle Outcome Score (FAOS) PROM: Short-Form (SF) 36 Complications
Re-operations

### Sports and clinical outcomes

Sports outcomes and its definitions are described in Table 2. Concerning the clinical outcomes, it was chosen to divide these into PROMs and other clinical outcomes. The numeric rating scale (NRS) for pain is a scale ranging from 0 (no pain) to 10 (worst pain imaginable). This questionnaire was assessed for pain in rest, during walking, during running and during stairclimbing [19]. The Foot and Ankle Outcome Score (FAOS) (Dutch version) is a validated questionnaire with 5 subscales: symptoms, pain, activities of daily living (ADL), sports and recreational activities, and ankle-related quality of life (QoL). All subscales range from 0 to 100 (0 indicating extreme symptoms and 100 indicating no symptoms) [20]. The SF-36 (Dutch version) has been validated in Dutch for the quality of life and is divided in eight different subscales with each scale score ranging from 0 to 100 (higher score equals less disability). The eight subscale scores can be merged into two summarized scores: the physical component summarized score (PCSS) and the mental component summarized score (MCSS) [1]. All numbers will be reported with two significant figures. Patients were asked to answer the questions based on the last seven days at the time of completing the questionnaire. Other clinical outcomes consisted of number and type of complications and re-operations and were extracted from the electronic patient system.

### Statistical analysis

Continuous outcome measures were presented as mean with standard deviation for data with normal distribution and as median with interquartile ranges according to the Tukey’s hinge method in case of non-normal distribution. Figures for non-normal distributed results are shown as boxplots. The boxplots show the median, the IQR and the minimum and the maximum. The IQR was described according to the Tukey’s hinge method. Categorical data were presented as frequencies with associated percentage. Normality was checked by the use of the Kolmogorov–Smirnov test and by the use of the Shapiro–Wilk test. Statistical analyses were performed with Statistical Package for Social Sciences (SPSS) version 26.0 (SPSS Inc. Chicago, IL). Due to the retrospective nature of the study and one moment of follow-up in time in combination with identifying all patients being suitable for inclusion, no sample size or (post hoc) power calculation was performed for this study as all potentially suitable patients that were able to be included were included for this study.

## Results

### Patient cohort

**Fig. 3:**
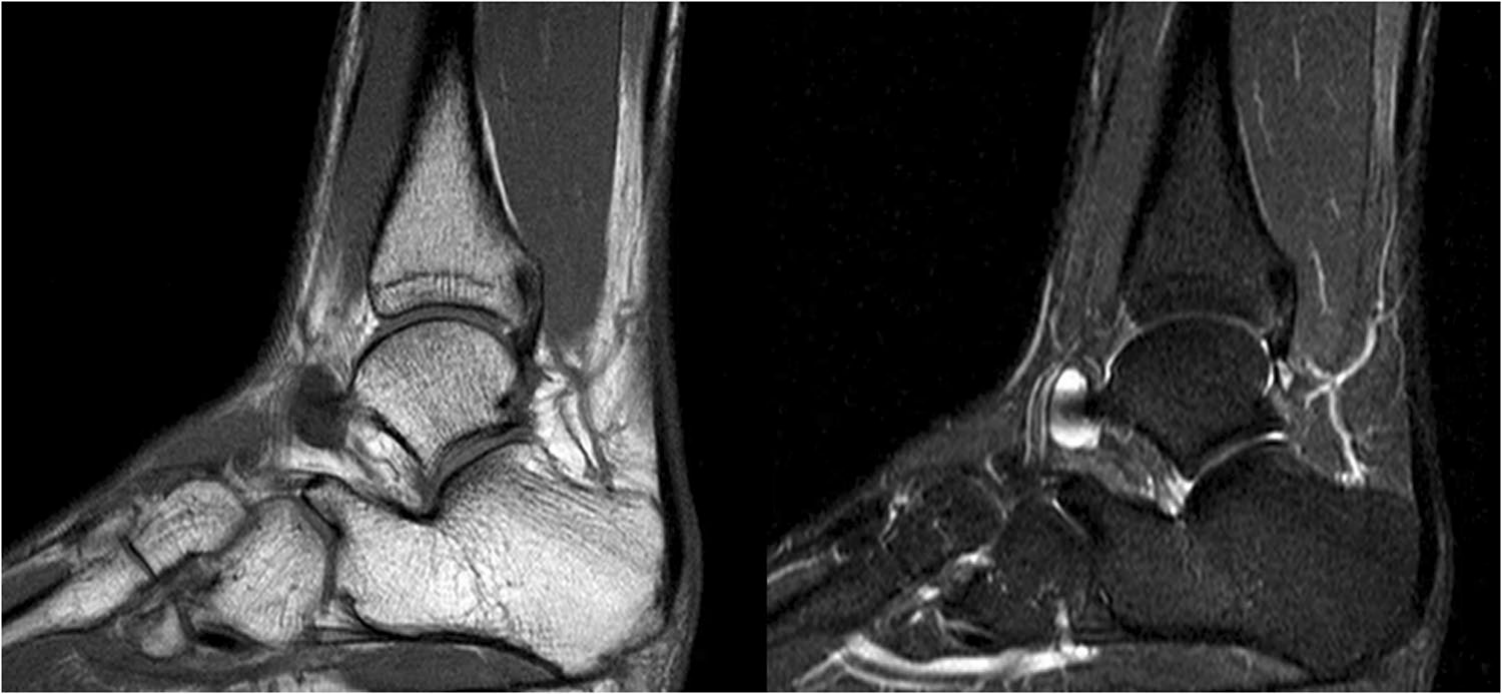
Sagittal MRI Coupes of a ganglion cyst. A ganglion cyst of 1.7 × 1.1 × 0.6 cm on the anterolateral side of the talar neck originating from the sinus tarsi (Left: T-1 Weighted, Right: Short-TI Inversion Recovery (STIR))

**Fig. 4:**
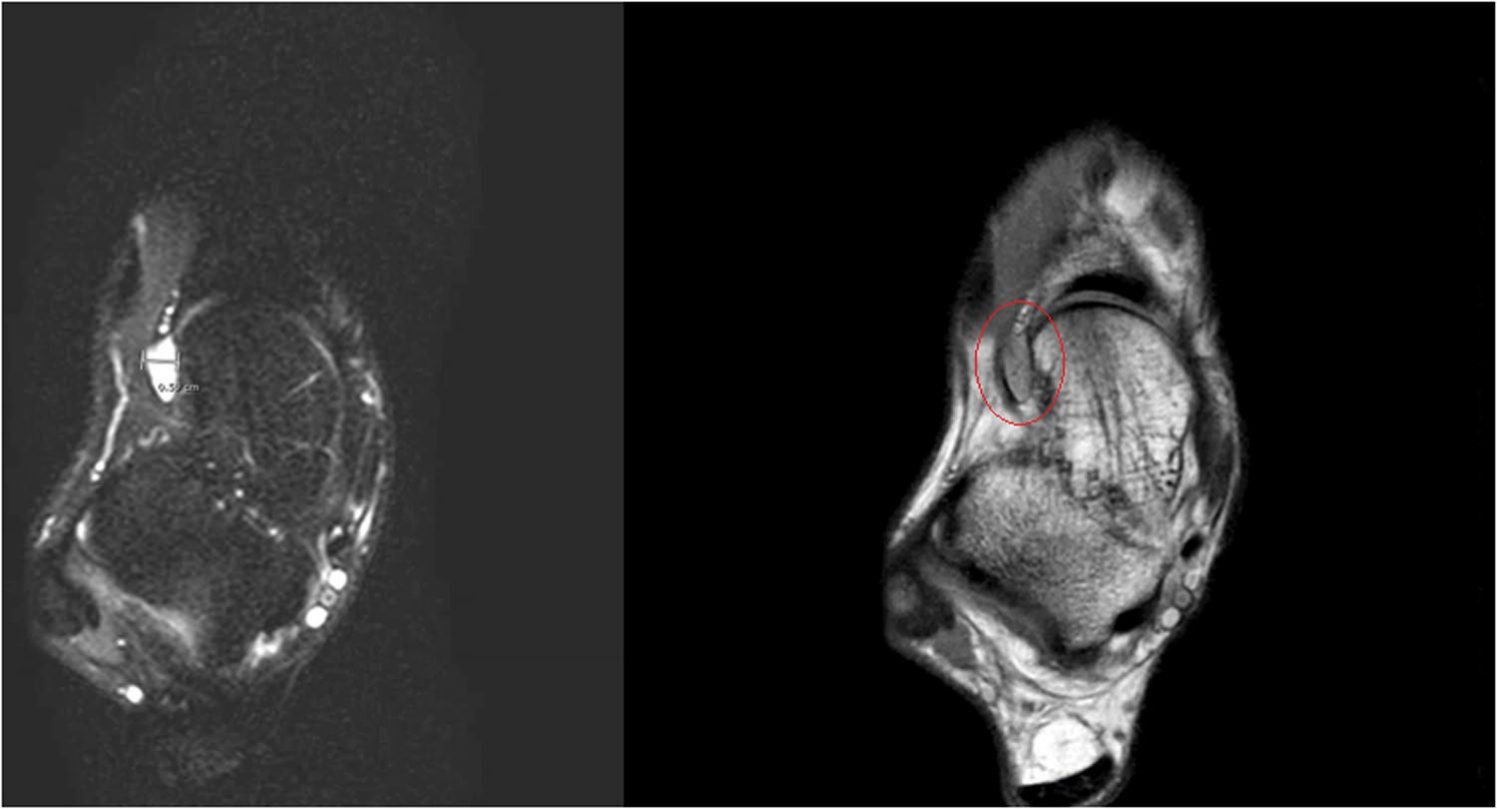
Axial MRI of a ganglion cyst. A ganglion cyst of 1.7 × 1.1 × 0.6 cm on the anterolateral side of the talar neck originating from the sinus tarsi (Left: T2 SPAIR Image (SPectral Attenuated Inversion Recovery), Right: Proton-Density (PD) Image)

All 22 patients (100%) responded to the phone call for the sports outcomes while 81% of the patients were able to complete the online clinical questionnaires through Castor EDC (Fig. 1). Table 3 shows the different indications for surgery being determined pre-operatively and confirmed intra-operatively. Figures 3 and 4 show the pre-operative MRI images of the ganglion cyst indication causing sinus tarsi syndrome.

**Table 3:** Reported indications for subtalar arthroscopy of the patients diagnosed with sinus tarsi syndrome

Indications	No. of patients (*N* = 22)
Soft tissue impingement	14
Osseous impingement	3
Corpus liberum	2
Synovitis	2
Ganglion	1

### Sports outcomes

In this study group, all patients were active in sports before surgery. Five patients (23%) practiced their sport on a professional level prior to the occurrence of the injury. Nine patients (41%) were active on a competitive level and eight patients (36%) exercised their sport on recreational level before the injury. The primary outcome of this study is the ‘return to pre-injury type of sport’: a total of twelve patients (55%) returned to their pre-injury type of sport at 23 weeks (median; IQR 10–45 weeks) postoperatively. The other sports outcomes are presented in Table 4.

**Table 4:** Sports outcomes of 22 patients, described in frequency and percentage

Sports outcomes	No. of patients	%	Return to sport time (weeks)
Return to any type of sport	21	95	12 (IQR 4.0–26)
*Return to pre-injury type of sport*	*12*	*55*	*23 (IQR 10–45)*
Return to performance	4	18	20 (IQR 8.0–46)
Improved performance	1	5.0	28

Italics represents primary outcome

### Clinical outcome measures

#### Numeric Rating Scale (NRS) of Pain

**Fig. 5:**
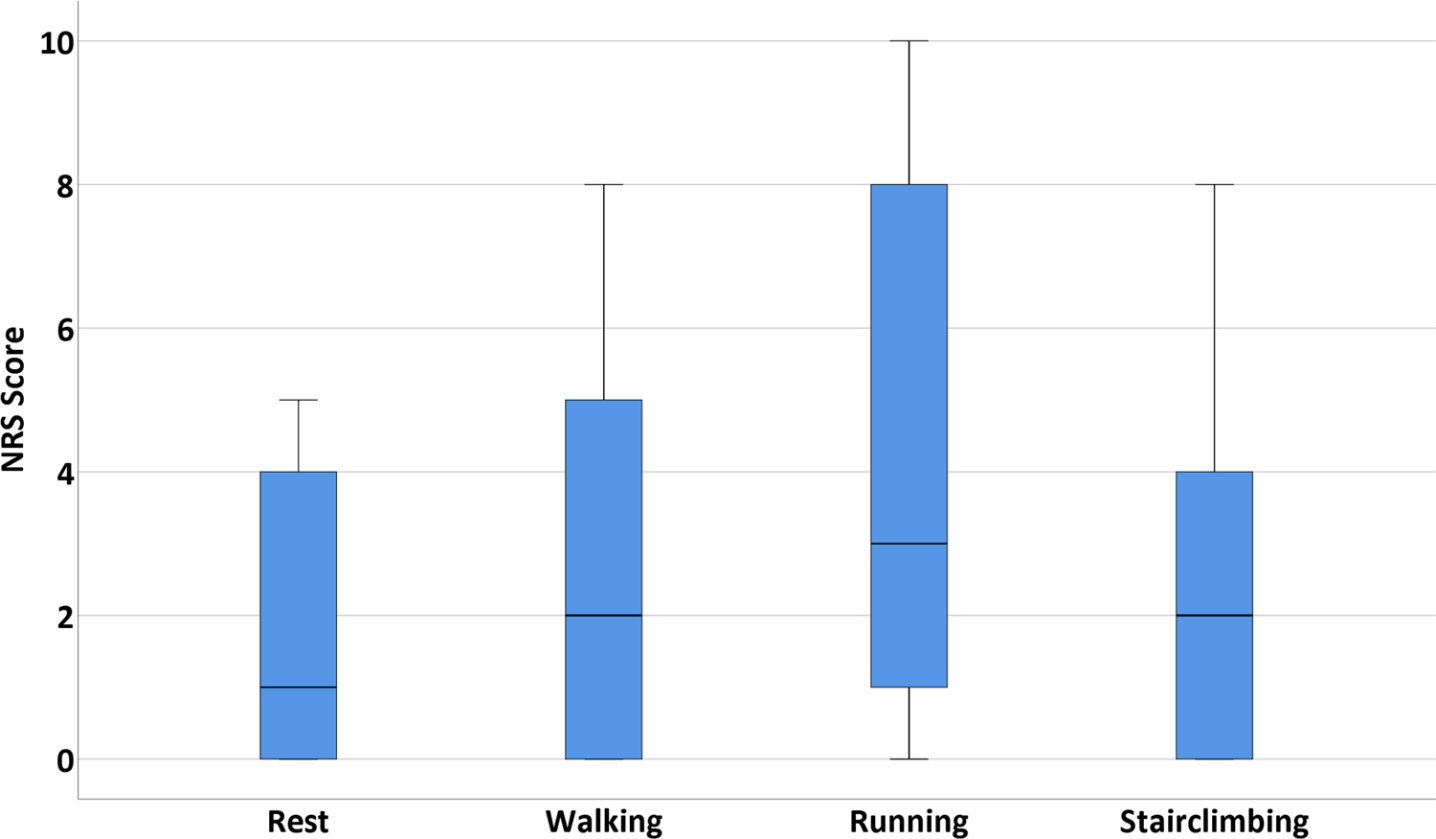
Postoperative Numeric Rating Scale (NRS) pain scores. NRS pain scores during four different situations scored by 18 patients diagnosed with sinus tarsi syndrome and treated with a subtalar arthroscopy

The median NRS score in rest was 1.0 (IQR 0.0–4.0) was noted (Fig. 5). During walking, the median number was 2.0 (IQR 0.0–5.0) on the NRS-scale. Patients experienced a median pain score of 3.0 (IQR 1.0–8.0) during running, and for stair climbing, a median NRS-score of 2.0 (IQR 0.0–4.0) was assessed.

#### Foot and Ankle Outcome Score (FAOS)

**Fig. 6:**
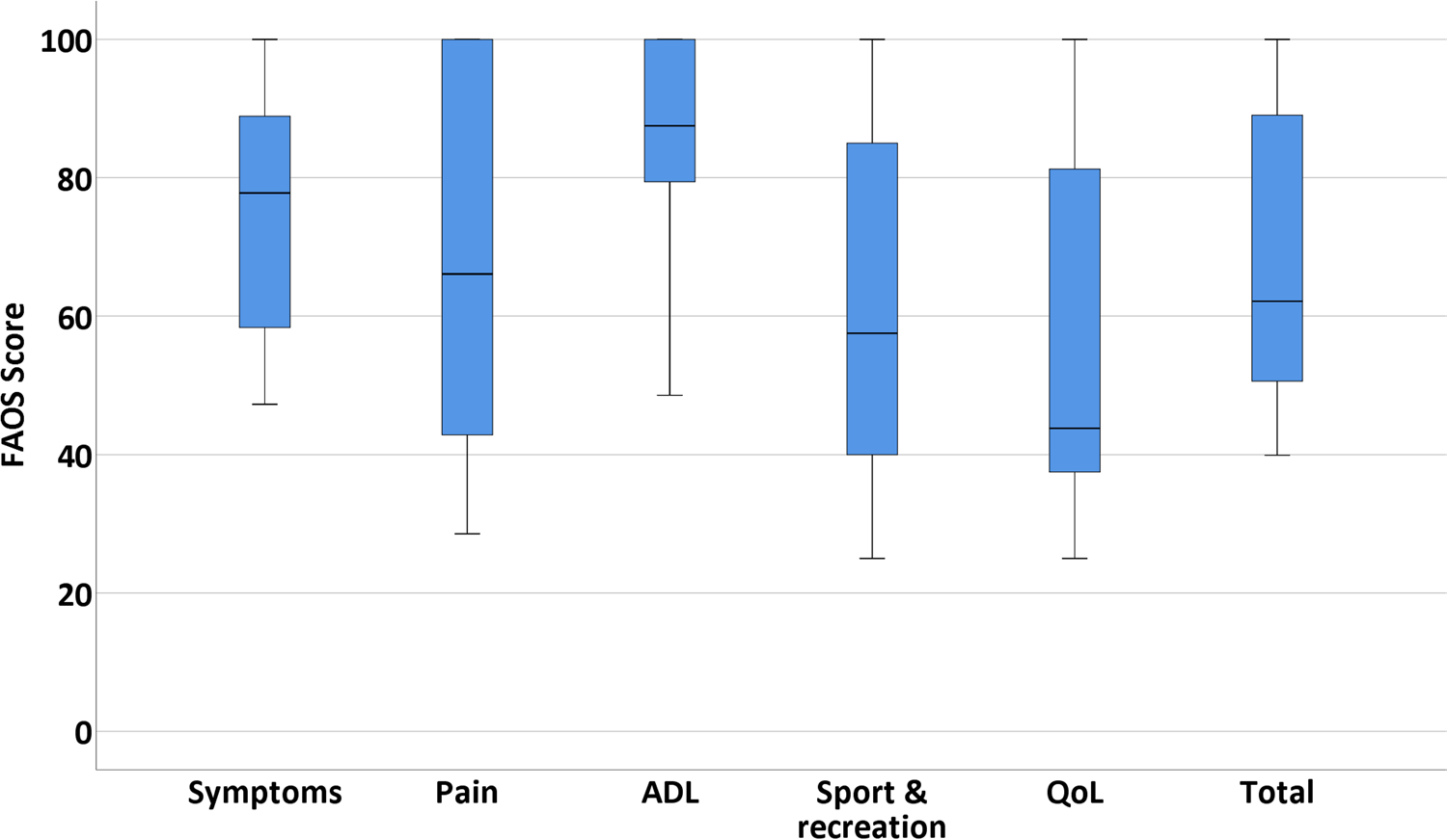
The Foot and Ankle Outcome Score (FAOS). FAOS of 18 patients, divided in five subscales and the total FAOS score

For the symptoms, a median of 78 was scored (IQR 58–89), and for the subscale of pain, the median was 66 (IQR 43–100) (Fig. 6). The median score in the subscale ADL was 88 (IQR 79–100). In sport and recreational activities, a median of 58 was found (IQR 40–85), and for the foot and ankle quality of life subscale, the median score was 44 (IQR 38–81). The median of the total score was 62 (IQR 51–89).

#### Short Form 36 (SF-36)

**Fig. 7:**
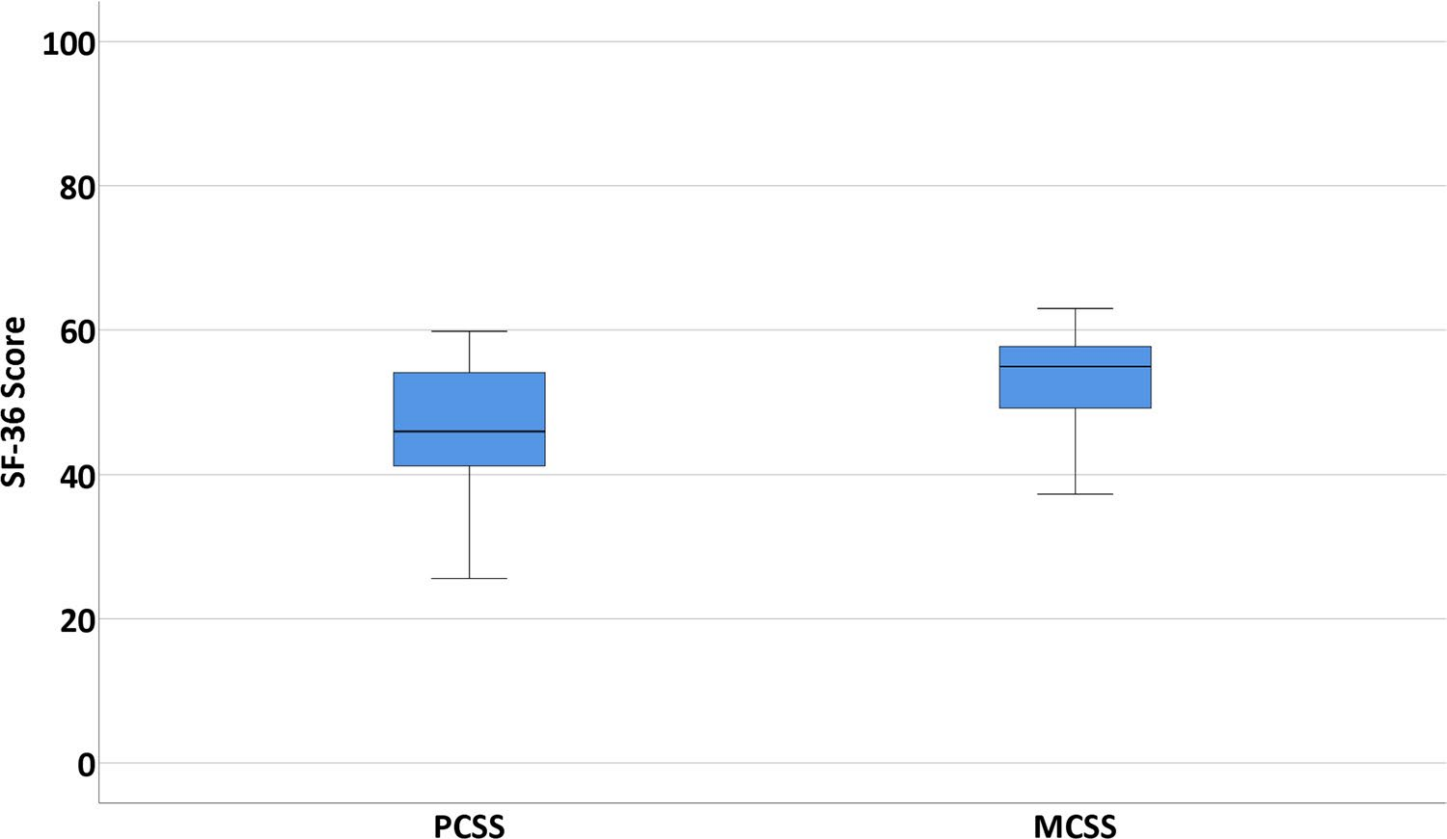
The 36-item Short Form Survey score (SF-36). The SF-36 score is divided into two subscales, the Mental Component Summarized Score (MCSS) and the Physical Component Summarized Score (PCSS)

For the PCSS, a median of 46 was calculated (IQR 41–54), the median of the MCSS subscale was 55 (IQR 49–58) (Fig. 7).

#### Complications and re-operations

There were no complications reported in the present study. One re-operation was reported (re-do subtalar arthroscopy). The patient experienced similar pain complaints 2 years after index surgery. The indication for the index surgery was performed due to osseous impingement as a consequence of a talar fracture. During the re-operation, soft-tissue impingement was diagnosed which was resolved through a nettoyage procedure. There were no pain complaints post-operatively, and after four weeks, the patient returned to cycling.

## Discussion

The most important finding of our study was that six out of ten patients returned to pre-injury type of sport after subtalar arthroscopy for sinus tarsi syndrome. Other important findings are that the present study shows that this specific intervention yields effective clinical and other sports outcomes, as assessed with adequate PROMs, return to sport data and safety outcome measures.

To the best of our knowledge, this is the first study publishing sports outcomes after subtalar arthroscopies as a treatment option for sinus tarsi syndrome. When considering return to sports outcomes for both the athlete as well as for academic purposes, it can be stated that return to sport should optimally be defined as a continuum. In a consensus statement published by Ardern et al. [2] in 2016, the continuum was defined in the order of ‘return to participation’, ‘return to sport’ and ‘return to performance’. In our study, return to participation was not used as a primary nor secondary outcome measure. The pre-injury type of sport as primary outcome measure in our study may be compared to the definition of the general return to sport in the consensus statement as posted by Ardern et al. [2] The definition of returning to performance in our study corresponds with the consensus statement being equal to the last part of the return to sport continuum. On top of the return to sport continuum as published by the team of Ardern et al. [2], another category was added for patients having improved their performances after the subtalar arthroscopy—this category was defined as returning to improved performance.

When analyzing these different sports outcome measures, it was clear that almost all (95%) patients returned to any sports at any level in the present patient population, while around six out of ten patients were able to return to their pre-injury type of sports. The return to performance and improved performance rates were lower, corresponding to rates of 18 and 5%, respectively. A number of different reasons were provided by the patients when qualitatively analyzing the failure to return to same pre-injury level or type of sport. The patients expressed that persisting pain in the ankle and suffering from concomitant injuries or comorbidities were common reasons for not returning to pre-injury level and/or type of sport. For a few patients who played their sport on a recreational level, it was a deliberate choice to quit with sport as they wished to not take the risk to injury the ankle again.

The return to sport times ranged from 12 to 28 weeks when analyzing the different levels of return to sport. It could be stated that these return to sport times and rates are adequate in comparison to other foot and ankle sports-related injuries, such as when considering osteochondral lesions of the talus, Lisfranc injuries, and syndesmotic injuries, for example [8, 14, 21, 24]. Steman et al. [21] found a mean rate of 88% for RTS at any level in patients treated for osteochondral lesions of the talus treated with arthroscopic bone marrow stimulation, while a RTS at pre-injury level and type of sport of 79% was found for the same surgical intervention. Lerch et al. [14] showed in their meta-analysis that around 67% of the active patients having suffered from an acute acute Achilles tendon rupture were able to return to their previous sporting level as being assessed by the Tegner Activity Scale [23, 25]. One can, therefore, note that the return to sport rates at the different levels are comparable with more common sports-related foot and ankle injuries, though it must be stated that it is difficult to assess the aforementioned outcomes in context of the current literature as clinical papers on sports outcomes after subtalar arthroscopy or open surgery for sinus tarsi syndrome are currently absent.

It was assessed that the patients reached a post-operative NRS during walking and running of 2.0 and 3.0, respectively, which shows the clinical efficacy of the procedure. This is in line with current literature on arthroscopic treatment [3, 12, 17]. Another clinical score that was measured in the present study was the Foot and Ankle Outcome Score (FAOS). An overall AOFAS score may be compared to the FAOS subscale of symptoms and daily functioning. When comparing these scores to the articles having been published on arthroscopic subtalar treatment for STS, similar clinical efficacy is proved [3, 12, 17, 18]. Both the pain outcomes (NRS) and other clinical PROMs of our study should however be interpreted in the light of length of follow-up of the different studies in the literature. Our study was performed at a median follow-up time of 60 months, while the different studies had a considerably shorter follow-up time (ranging from 12 to 24 months) than our data being presented making the present study—to the best of our knowledge—the study with the longest follow-up in the highest number of patients in the literature [12, 17, 18].

Concerning clinical safety and complication data, it was shown that one re-operation was recorded and that there were no complications. Other studies reported, respectively, five, one and no complication, respectively, varying from a superficial wound infection to an irritation of the lateral branch of the superficial peroneal nerve. Complete resolution of symptoms was shown after neurolysis procedure for the lateral branch of the superficial peroneal nerve. Furthermore, all other complications resolved with conservative therapy [6, 12, 18]. This assumes that the subtalar arthroscopy as a whole can be considered a safe treatment procedure.

The injury of sinus tarsi syndrome can be considered a continuum concerning its treatment paradigms. First, a conservative algorithm is initiated and is considered to be effective in up to 63% of patients [10, 22]. For patients with persisting complaints of sinus tarsi treatment after 1 year of intense conservative treatment, the next stage in the clinical continuum is reached—a stage which is considered difficult to treat amongst foot and ankle surgeons and sports medicine physicians. The present paper focuses on that stage, and although our results show clinical and sports effectiveness, the majority of the foot and ankle orthopaedic surgeons decide to perform a subtalar arthrodesis, thereby resolving the patients’ pain. A specific disadvantage of this technique is however the permanent limitations in range of motion movement of the subtalar joint.

The present study needs to be interpreted in its strengths and limitations. As noted earlier, our study is the first study performed analyzing sports outcomes for subtalar arthroscopies as treatment option for sinus tarsi syndrome. Moreover, it is the study with the longest follow-up time concerning all studies on subtalar arthroscopy for STS. Additionally, no patients were lost to follow-up, and there was a 100% completion rate obtained for our primary outcome. Despite an 83% completion rate for our secondary clinical outcomes, it is clear that this lower completion rate did not influence the primary outcomes of the study. Limitations of the study are the absence of pre-operative scores. Hence, we were not able to calculate whether the minimal clinical important difference (MCID) for the different scores were reached. Due to the retrospective nature of the study and one moment of follow-up in time in combination with identifying all patients being suitable for inclusion, no (post-hoc) power calculation could be performed, which should be mentioned as a limitation.

With the first framework of reference in sportive outcomes and the long-term clinical outcomes for subtalar arthroscopies as treatment for sinus tarsi syndrome, this study can be considered of high clinical relevance. For the treating physician and the supporting team, the effects of subtalar arthroscopies on sports and long-term clinical outcomes have been clarified through the execution of the present study. It can be stated that athletic patients being diagnosed with sinus tarsi syndrome may consider subtalar arthroscopy as a treatment option with appropriate and effective sports outcomes at long-term follow-up available in the current evidence. As a consequence, the results of the present study can be utilised in daily clinics to inform patients—especially active patients—and their relatives on the expected long-term return to sport rates and times at different levels of sports as well as long-term clinical outcomes. This will increase and ameliorate the quality and efficacy of the shared-decision making process for the different medical specialties treating patients with this syndrome, that is, physiotherapists, rehabilitation physicians, orthopaedic surgeons, sports medicine physicians as well as general practitioners.

## Conclusion

Six out of ten patients with sinus tarsi syndrome returned to their pre-injury type of sport after being treated with a subtalar arthroscopy. Furthermore, the outcomes of the present study show that subtalar arthroscopy yields effective outcomes at long-term follow-up concerning patient-reported outcome measures in athletic population, with favorable return to sport level, return to sport time, clinical outcomes and safety outcome measures. The results of the study can be used to inform patients—specifically active patients—about expected sports and clinical outcomes.

## Abbreviations

ADL: Activities of daily living
AOFAS: American Orthopaedics Foot and Ankle Society
AUMC: Amsterdam University Medical Centre
CL: Cervical ligament
CT: Computed tomography
FAOS: Foot and Ankle Outcome Score
GK: Prof. Dr. G.M.M.J. Kerkhoffs
IER: Inferior extensor retinaculum
IQR: Interquartile range
ITCL: Interosseous talocalcaneal ligament
MCID: Minimal clinical important difference
MCSS: Mental Component Summarized Score
MRI: Magnetic resonance imaging
NRS: Numerous Rating Scale
OCD: Osteochondral defects
PCSS: Physical Component Summarized Score
QoL: Quality of life
RF: Radiofrequency
RTS: Return to sport
SD: Standard deviation
SF-36: 36-Item short form survey
SS: Dr. S.A.S. Stufkens
STS: Sinus tarsi syndrome
VAS: Visual Analog Scale
